# Abnormal Sleep Duration as Predictor for Cardiovascular Diseases: A Systematic Review of Prospective Studies

**DOI:** 10.1155/2022/9969107

**Published:** 2022-02-07

**Authors:** Sidhi Laksono, Mefri Yanni, Mohammad Iqbal, Ananta Siddhi Prawara

**Affiliations:** ^1^Department of Cardiology and Vascular Medicine, RS Pusat Pertamina, Faculty of Medicine, Universitas Muhammadiyah Prof. DR. Hamka, Tangerang, Indonesia; ^2^Department of Cardiology and Vascular Medicine, RSUP M. Djamil, Faculty of Medicine, Universitas Andalas, Padang, Indonesia; ^3^Department of Cardiology and Vascular Medicine, RSUP Hasan Sadikin, Faculty of Medicine, Universitas Padjadjaran, Bandung, Indonesia; ^4^Faculty of Medicine, Universitas Diponegoro, Semarang, Indonesia

## Abstract

**Methods:**

We searched the online database PubMed on 30 August 2020 for our data collection. We used the following keywords: sleep duration AND (cardiovascular disease OR cardiovascular event) AND (cohort OR prospective OR retrospective). We identified 653 studies, and after excluding studies that were published before 2015, we obtained 306 studies. After filtering the 306 studies through title and abstract screening and applying the inclusion and exclusion criteria, we further reviewed fourteen studies with full-text reading. We excluded three studies because of insufficient data required and included eleven studies in this systematic review.

**Results:**

A total of 361,041 participants from ten studies were included in this systematic review. The incidence of hypertension, myocardial infarction, coronary artery disease, heart failure, cardiovascular events, and cardiovascular diseases in the short sleep duration group is 46.12%, 0.59%, 5.43%, 0.09%, 7.18%, 1.48%, and 6.8%, consecutively, while the incidence of hypertension, myocardial infarction, coronary artery disease, and heart failure in the long sleep duration group is 30.71%, 0.61%, 6.55%, 1.11%, and 6.04%, consecutively. Nine studies reported an association between sleep duration and cardiovascular diseases while one study reported no association. Seven studies reported that short sleep duration was significantly associated with CVD. Short sleep duration in this study was associated with hypertension and heart failure. Atrial fibrillation and coronary artery disease were associated with both short and long sleep duration.

**Conclusion:**

Abnormal sleep duration (short and long sleep duration) may act as the predictor of cardiovascular diseases. The importance of having normal sleep duration should be stressed with other lifestyle modification to avoid the risk of getting cardiovascular diseases. However, further studies are needed to overcome the limitation of this systematic review.

## 1. Introduction

Cardiovascular diseases, a group of disorders of the heart and blood vessels, were responsible for the highest death rate in the world. It was estimated to be the cause of death of 17.9 million people in 2017 [1]. The advancement of cardiovascular diseases' management that can be seen in the recent guidelines published by the European Society of Cardiology (ESC), American College of Cardiology (ACC), and International Society of Hypertension (ISH) brings hope to reduce the morbidity and mortality of the patients with cardiovascular diseases. The importance of lifestyle modifications (including balanced diet, physical activity, and obstructive sleep apnea management) has been stressed on the published guidelines [2–10]. However, the significance of having enough sleep was not yet included in the guidelines, even though it might further help the patients, especially in preventing further cardiovascular diseases.

The prevalence of people with a sleep disorder or report insufficient sleep has increased; it was reported by the National Heart, Lung, and Blood Institute of the National Institutes of Health that approximately 50 to 70 million American adults suffer from sleep disorder [11]. The increasing prevalence of people with abnormal sleep duration may be caused by an increase in activities that operate 24/7 and usage of electronics such as mobile phones [12]. More activities that operate 24/7 resulted in more workers working under shift works. Different shift works (morning, afternoon, and night shifts) have different impacts to the quantity and quality of sleep as described by Åkerstedt [13], while the increasing use of electronics including mobile phones during the day and bedtime have a negative correlation with sleep duration [14]. The American Academy of Sleep Medicine (AASM) and Sleep Research Society (SRS) published a consensus in 2015 which defined normal sleep duration as sleep 7 or more hours per night. However, the consensus also stated that sleeping more than 9 hours per night was associated with health risk for those who were not included in one of these: young adults, individuals recovering from sleep debt, and individuals with illness [15]. Abnormal sleep duration, especially short sleep duration, was known to increase the sympathetic tone from the predominant neutral interaction of sympathovagal. These changes in autonomic dysregulation led to cardiovascular diseases [16]. In another study, it was also suggested that abnormal sleep duration may alter circadian clock genes which acted as a central regulator of the circadian rhythm [17].

Circadian rhythm in the human body is generated by two major clocks: the central clock and the peripheral clock. The central clock refers to the hypothalamic master clock in the suprachiasmatic nucleus (SCN). Changes of light, which was detected by the retinal cells of the eyes, signal the central clock to activate the transcription of *Clock* (circadian locomotor output cycles kaput) and *Bmal1* (brain and muscle Arnt-like protein 1) genes. These genes played an important role in activating molecular sequences of the circadian rhythm [18]. On the other hand, the peripheral clock refers to the intrinsic circadian rhythms in each organ system, including the cardiovascular system, which may act independently from the central clock [19]. The circadian mechanism can be regulated in the absence of light/dark cycles by food intake, temperature, and environment. The environmental stimuli may affect circadian rhythm specifically according to the cells and tissues; they were known as zeitgebers. The zeitgebers also regulate circadian rhythm in cardiomyocytes, fibroblasts, and vascular smooth muscle cells [18]. Alterations of central and/or peripheral circadian rhythm because of abnormal sleep duration may cause abnormality in hormonal secretion, blood pressure, heart rate, endothelial cell function, platelet aggregation, thrombus formation, and other physiological processes [18, 20]. Thus, we are aiming to figure out the role of abnormal sleep duration in predicting cardiovascular diseases. Our systematic review will provide a better evidence regarding abnormal sleep duration and cardiovascular outcomes. There are no systematic review regarding abnormal sleep duration and hypertension.

## 2. Methods

This review has been reported following the Preferred Reporting Items for Systematic Reviews (PRISMA) guidelines 2015. We used population (patient with sleep disorder), intervention/exposure (sleep disorders), comparator (without sleep disorders), outcome (cardiovascular disease), and study design (cohorts) using the PICOs framework to formulate the research question.

### 2.1. Inclusion and Exclusion Criteria

We evaluated English language papers published within five years that evaluate the association between sleep duration and cardiovascular diseases. We included only prospective cohort studies that were previously peer reviewed and all participants that did not have prior specific cardiovascular diseases that were being measured by each study included. We excluded surveys, cross-sectional studies, case-control studies, and Mendelian randomization.

### 2.2. Types of Outcome Measurement

The measurement of sleep duration could be done objectively or subjectively. Sleep duration is divided as short and long sleep duration. Short sleep duration was defined as sleep less than or equal to 4.9 hours to less than 7 hours, while long sleep duration was defined as sleep more than or equal to 7.5 hours to more than or equal to 10 hours. Questionnaires or interview are considered as subjective measurement of sleep duration. The performance of subjective measurement of sleep duration was known to vary according to demographic and did not closely correspond to the objective measurement of sleep [19, 20]. However, this method was more efficient and practicable in collecting sleep duration data in a large population study, which suited the condition of the 10 studies [21]. Sleep duration was assessed with questionnaires in 9 studies and interviews in 2 studies by asking how many hours they slept on average during the month in one study and how many hours they slept usually in a day in another. Short sleep duration and long sleep duration were defined differently in the studies. Short sleep duration was defined as sleep less than or equal to 4.9 hours to less than 7 hours, while long sleep duration was defined as sleep more than or equal to 7.5 hours to more than or equal to 10 hours.

The primary outcome included in this study is the duration of sleep and cardiovascular disease incident in patients that have no prior specific cardiovascular diseases that was evaluated by each study. The secondary outcomes included in this study are the number of participants, follow-up duration (assessed from follow-up information provided in the study), participants' age (assessed from reported age information in the study), male percentage (assessed from reported numbers of male in the study divided by the total participants), and sleep assessment tool used (assessed from information regarding tools being used in the study).

### 2.3. Electronic Search

Two independent authors searched the online database PubMed on 30 August 2020 for our data collection. We used the following keywords: sleep duration AND (cardiovascular disease OR cardiovascular event) AND (cohort OR prospective OR retrospective).

### 2.4. Study Selection

**Figure 1:**
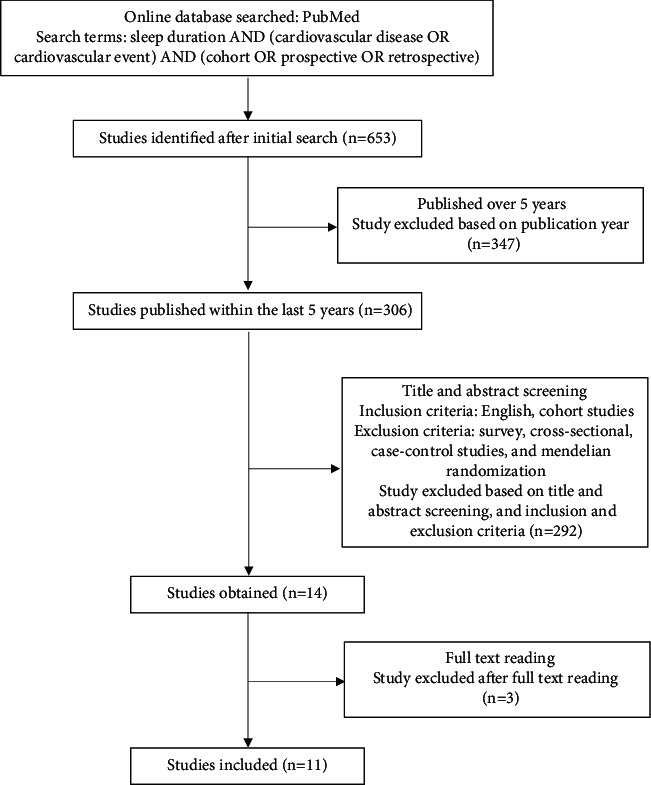
Study consort diagram of the systematic review.

653 studies were identified, and after excluding studies that were published before 2015 (latest study, study from the last 5 years), 306 studies were obtained. After filtering the 306 studies through title and abstract screening and applying the inclusion and exclusion criteria, thirteen studies were further reviewed with full-text reading. Three studies were excluded because of insufficient data required. Thus, ten studies were included in this systematic review. The study consort diagram can be seen in Figure 1. Two authors (SL and ASP) independently evaluated the title and abstracts of the retrieved articles. The selected articles in the first step of evaluation had their full text assessed by the same reviewers. In cases of any disagreements in the previous steps, decision will be taken after discussion. We used the Newcastle-Ottawa quality assessment scale (NOS) for cohort studies to evaluate the quality of included studies. In the current study, we considered a study awarded six or more points as a high quality study. Discrepancies were resolved by discussion. In this study, 3 studies are classified as high risk and 7 studies as high-quality studies. Follow-up varies from 3 to 20 years.

### 2.5. Data Extraction

Two authors (SL and ASP) also performed data extraction and study quality assessment independently. Data extraction was organized in spreadsheets. The extracted data of each study included the following information: the authors' name, year of publication, country, number of participants, baseline year, follow-up duration, mean participants' age, male percentage, sleep assessment tool, outcome assessed, sleep category, normal sleep duration, the result (incidence, OR/HR), conclusion, and adjusted variables. The meta-analysis was not feasible due to different definitions of sleep disorder; this different definition caused a different exposure too.

## 3. Results

A total of 361,041 participants from 10 prospective cohort studies published between 2015 and 2020 were included in this systematic review. The studies included were conducted in 6 countries and 3 continents. The cardiovascular diseases assessed by the 10 studies differed; 3 assessed hypertension, 1 assessed myocardial infarction, 2 assessed coronary heart disease, 1 assessed atrial fibrillation, 1 assessed heart failure, 1 assessed cardiovascular disease (myocardial infarction, angina pectoris, revascularization procedure, and stroke), and 1 assessed cardiovascular events (atrial fibrillation, myocardial infarction, and stroke). The baseline information of the studies can be seen in Table 1.

The 9 studies included in this systematic review reported an association between sleep duration and cardiovascular diseases while 2 studies reported no association. From the 9 studies which reported the association, 7 reported that short sleep duration was significantly associated with CVD. Short sleep duration in this study was associated with hypertension, coronary heart disease, and cardiovascular events (atrial fibrillation, myocardial infarction, and stroke). Odds ratio from study being used in this review varies from 0.89 to 3.15. Most of the studies could not control all of confounders, while some control few of its confounders. On the other hand, 2 studies reported that long sleep duration was significantly associated with CVD. The CVDs that were associated with long sleep duration were atrial fibrillation and coronary heart disease (nonfatal MI, stable angina, unstable angina, unspecified CHD, or CHD death). Hypertension was not cited as exclusion criteria in 8 studies which are aimed at finding an association between sleep duration and the occurrence of other cardiovascular diseases. However, we still included the 8 studies because blood pressure or hypertension was still included as one of the adjusted variables when the studies find the association between sleep duration and cardiovascular disease. The association between sleep duration and cardiovascular disease from 10 studies can be seen in Table 2.

## 4. Discussion

Overall, the evidence might suggest association between abnormal sleep duration and cardiovascular outcome. Three studies show short sleep duration was consistently associated with the occurrence of hypertension in middle-aged participants (both men and women) [22–24]. Four included studies reported that two cardiovascular diseases, coronary heart diseases and atrial fibrillation, are associated with both long and short sleep duration. Two studies in this systematic review assessed a more general outcome which was cardiovascular events and cardiovascular diseases [25–29].

### 4.1. Abnormal Sleep Duration and Cardiovascular Diseases

Short sleep duration was consistently associated with the occurrence of hypertension in middle-aged participants (both men and women) from the three studies [22–24]. The study conducted by Mallinson et al. specifically mentioned that the occurrence of hypertension in women was higher [23]. This finding was supported by a previous study conducted by Bertisch et al. which also stated that short sleep duration in women was associated with hypertension compared to women who have normal sleep duration [30]. Align with hypertension, coronary artery calcification, and heart failure were associated with short sleep duration [28, 31]. Multiple mechanisms, not one, are responsible for the development of cardiovascular diseases from short sleep duration. The mechanisms are the increase of proinflammatory cytokines, sympathetic activity, oxidative stress, hypercholesterolemia, ghrelin, and the decrease of leptin. These mechanisms may be linked to circadian misalignment directly or indirectly [32].

Four studies have reported that two cardiovascular diseases, coronary heart diseases and atrial fibrillation, were found to be associated with both long and short sleep duration [25–28]. Tobaldini et al. found that fibrinogen may be responsible as the mechanism underlying the coronary heart disease occurrence in participants with long sleep duration even though the exact mechanism is not understood yet [33]. A systematic review that was aimed at figuring out the association of atrial fibrillation and sleep duration conducted by Morovatdar et al. found a similar result with the findings in this systematic review in which atrial fibrillation was associated with short and long sleep duration. However, the authors mentioned that the exact mechanism between atrial fibrillation and sleep duration remains unclear [34]. In a different study, it was found that sleep deprivation caused a reduction in left atrium early diastolic strain rate, which in the long term may develop into atrial fibrillation [35]. Generally, long sleep duration can be associated with an increased risk of CVD because long sleep duration itself was associated with depressive symptoms, low socioeconomic status, unemployment, and low physical activity. These conditions that were associated with long sleep duration can also act as CVD risk factors [36].

Myocardial infarction was not reported to be associated with abnormal sleep duration in a study conducted by Song et al. [37]. The association was also not found when myocardial infarction was being assessed as a component of cardiovascular diseases, except when the participants have short sleep duration and insomnia [29]. The association of myocardial infarction and sleep duration was found when myocardial infarction was assessed as a component of either coronary heart disease [25] (long sleep duration) or cardiovascular events [28] (low stable pattern).

Two studies in this systematic review assessed a more general outcome which was cardiovascular events and cardiovascular diseases. Cardiovascular events were stated to be associated with low stable sleep pattern, followed by low increasing sleep pattern. These findings suggest that despite the follow-up sleep duration, the short sleep duration in the baseline can predict cardiovascular events. However, the finding can also be interpreted that the risk of the occurrence of cardiovascular events can be reduced by increasing sleep duration [28]. The association of short sleep duration and cardiovascular diseases was not found in the study conducted by Wang et al., but the association was found when assessing the relation between patients with short sleep duration and insomnia/poor sleep with cardiovascular diseases [29]. This finding suggested that besides sleep duration, altered sleep quality (insomnia/poor sleep) may also affect cardiovascular diseases.

The findings from the studies included in this systematic review suggest that both short sleep duration and long sleep duration may be associated with cardiovascular diseases. A previous study conducted by Kwok et al. has confirmed that there was a U-shaped relation between sleep duration and hypertension [38]. Guo et al. also found a U-shaped relation for CHF, stroke, and coronary artery disease with sleep duration in their study [39].

### 4.2. Normal Sleep Duration

The 10 studies defined normal sleep duration differently. Four studies defined 7 hours as normal sleep duration. Seven other studies defined normal sleep duration using range: >6 hours, 6-7.9 hours, 6-8 hours, 7-7.9 hours, 7-<7.5 hours, 7-<8 hours, and 7.4-7.5 hours. The differences of normal sleep duration of the 10 studies may be due to the baseline year of when the studies began before the publication of the consensus.

### 4.3. Study Limitations

There are several limitations to this systematic review. First, normal sleep duration was defined differently in the studies included in this study. The first limitation limited us on performing a meta-analysis. Second, sleep duration in the studies included in this systematic review was measured subjectively. Objective measurements can be considered in future studies. Third, we did not assess the occurrence of obstructive sleep apnea in this systematic review. Fourth, besides sleep duration, sleep patterns and sleep quality can also affect the cardiovascular system. Thus, a further study explaining sleep patterns and sleep quality is needed to complete the information about sleep that can be given to the patients.

## 5. Conclusion

Both short and long sleep duration was associated with cardiovascular diseases compared to normal sleep duration. Short sleep duration was associated with hypertension, coronary artery calcification, and heart failure. Coronary heart disease and atrial fibrillation can be associated with both short and long sleep duration. The importance of having normal sleep duration should be stressed during educating the patients, besides other lifestyle modifications. Further studies are needed to overcome the limitation of this systematic review.

## Figures and Tables

**Table 1:** Baseline information of the 10 prospective cohort studies.

No.	Authors	Year of publication	Country	Number of participants	Baseline year	Follow-up (years)	Mean participants' age (years)	Male percentage (%)	Sleep assessment tool	Outcome assessed
1	Song et al. [37]	2015	China	95,903	2006-2007	Mean 3.98	51.33 ± 12.44	79.55	Questionnaire	Myocardial infarction
2	Girschik et al. [22]	2016	South Korea	1715	2005-2008	Mean 2.6	53.54	36.68	Questionnaire	Hypertension
3	Mallinson et al. [23]	2016	China	32,137	2006-2007	Mean 3.98	46.32 ± 11.50	73.41	Questionnaire	Hypertension
4	Yadav et al. [24]	2016	China	874	2011-2012	Mean 2	56.14	25.29	Interview	Hypertension
5	Wang et al. [25]	2016	China	19,370	2008-2010	3 to 5	62.8	30.11	Questionnaire	Coronary heart disease (nonfatal MI, stable angina, unstable angina, unspecified CHD, or CHD death)
6	Song et al. [31]	2016	UK	3723	2003	9	68.13	100	Questionnaire	Heart failure
7	Lao et al. [27]	2017	China	87,693	2006-2007	Median 7.89	50.54	78.65	Questionnaire	Atrial fibrillation
8	Wang et al. [29]	2017	USA	6441	1995-1998	Median 11.4	64.0 ± 11.1	46.5	Questionnaire	Cardiovascular disease (first event of nonfatal or fatal myocardial infarction, angina pectoris, revascularization procedure, or stroke)
9	Yang et al. [26]	2018	Taiwan	60,586	1996-2014 (for total cohort)	Mean 5.6	50.6 ± 8.6	46.3	Interview	Coronary heart disease
10	Wannamethee et al. [28]	2020	China	52,599	2006, 2008, 2010	Mean 6.7	49.0 ± 11.8	76.1	Questionnaire	Cardiovascular events (atrial fibrillation, myocardial infarction, and stroke)

**Table 2:** Association between sleep duration and cardiovascular diseases.

No.	Authors	Sleep category	Normal sleep duration	Outcome assessed	Incident of CVD and hazard ratio (HR)/odds ratio (OR)	Conclusion	Adjusted variables
1	Song et al. [37]	≤5, 6, 7, 8, and ≥9 hours	7 hours	Myocardial infarction	The number of cases of MI in those who slept ≤5 h was 40 out of 6736 (0.59%) and 10 out of 1641 (0.61%) for those who slept ≥9 hours. HR of MI in participants who slept ≤5 hours was 0.89 (95% CI: 0.60-1.30) and ≥9 hours was 1.12 (95% CI: 0.58-2.16).	There was no significant association between sleep duration and myocardial infarction.	Age, sex, family per member monthly income, education level, marital status, smoking status, drinking status, physical activity, history of hypertension, diabetes mellitus, and hyperlipidemia
2	Girschik et al. [22]	<6, 6-7.9, 8-9.9, and ≥10 hours	6-7.9 hours	Hypertension	164 participants developed hypertension (9.56%). OR for new-onset hypertension was 1.71 (95% CI: 1.01-2.89) with short sleep duration < 6 hours.	Short sleep duration was independently associated with the development of hypertension among the middle-aged and elderly.	Age, sex, education, smoking, alcohol, income, regular exercise, obesity, high-density lipoprotein cholesterol, triglyceride, glucose, and mean arterial pressure
3	Mallinson et al. [23]	≤5, 6, 7, 8, and ≥9 hours	7 hours	Hypertension	12,732 out of 32,127 participants developed hypertension. Short duration of sleep (≤5 h) was associated with increased hypertension in women (HR 1.27) (95% CI: 1.02-1.58) and participants aged <60 years (HR 1.11) (95% CI: 1.02-1.21).	Short sleep duration was associated with increased hypertension in women and participants aged <60 years.	Age, resting heart rate, body mass index, smoking status, drinking status, physical activity, salt intake, history of diabetes and hyperlipidemia, antidiabetic and cholesterol-lowering medication, systolic blood pressure, diastolic blood pressure, and family history of hypertension
4	Yadav et al. [24]	≤4.9, 5-5.9, 6-6.9, 7-7.9, and ≥8 hours	7-7.9 hours	Hypertension	24.8% (*n* = 217) developed hypertension. Among the younger age group 40-55 years, short sleep duration ≤ 4.9 h was associated with higher risk of hypertension (OR: 3.15) (95% CI: 1.04-9.54).	Short sleep duration was associated with higher risk of hypertension among participants with younger age (40-55 years old). No association was found among participants with older age (55-70 years old).	Sex, baseline blood pressure, personality, BMI, diabetes mellitus, physical exercise, smoking, drinking, total cholesterol, triglyceride, HDL-c, LDL-c, hs-CRP, uric acid
4	Song et al. [37]	≤5, 6, 7, 8, and ≥9 hours	7 hours	Myocardial infarction	The number of cases of MI in those who slept ≤5 h was 40 out of 6736 (0.59%) and 10 out of 1641 (0.61%) for those who slept ≥9 hours. HR of MI in participants who slept ≤5 hours was 0.89 (95% CI: 0.60-1.30) and that in participants who slept ≥9 hours was 1.12 (95% CI: 0.58-2.16).	There was no significant association between sleep duration and myocardial infarction.	Age, sex, family per member monthly income, education level, marital status, smoking status, drinking status, physical activity, history of hypertension, diabetes mellitus, and hyperlipidemia
5	Wang et al. [25]	<7, 7-<8, 8-<9, 9-<10, and ≥10 hours	7-<8 hours	Coronary heart disease (nonfatal MI, stable angina, unstable angina, unspecified CHD, or CHD death)	There are a total of 2058 incidents of CHD. There are 133 CHD incidents out of 1012 participants who slept for ≥10 h. The HR of CHD incidence for those who slept ≥10 h was 1.33 (95% CI: 1.1-1.62).	Longer sleep duration was associated with a higher risk of CHD incidence.	Age, sex, BMI, education, smoking status, drinking status, physical activity, hypertension, hyperlipidemia, diabetes, family history of CHD, and sleep duration
6	Song et al. [31]	<6, 6, 7, 8, and ≥9 hours	7 hours	Heart failure	There were 199 incident HF cases from 3723 men. Heart failure occurred in 25 patients out of 348 patients in those who reportedly slept less than 6 hours with HR 1.26 (95% CI: 0.77-20.5) after adjusting several factors.	Short sleep duration (<6 hours) in men was associated with high risk of developing heart failure.	Age, type of work, body mass index, smoking, diabetes mellitus, physical activity, treated hypertension, breathlessness, preexisting myocardial infarction, stroke, poor health
7	Lao et al. [27]	≤5, 6, 7, 8, and ≥9 hours	7 hours	Atrial fibrillation	322 cases (0.37%) of atrial fibrillation occurred. The short sleep duration (≤6 h) HR for atrial fibrillation was 1.07 (95% CI: 0.75-1.53) and long sleep duration (≥8 h) HR for atrial fibrillation was 1.50 (95% CI: 1.07-2.10).	Long sleep duration may be a potential predictor for the incident of atrial fibrillation.	Age, sex, education, smoking, alcohol, physical activity, snoring, body mass index, hypertension, diabetes mellitus, dyslipidemia, myocardial infarction, uric acid, and high-sensitivity C-reactive protein
8	Wang et al. [29]	<6 hours and normal sleep, insomnia/poor sleep or not	>6 hours	Cardiovascular disease (first event of nonfatal or fatal myocardial infarction, angina pectoris, revascularization procedure, or stroke)	14.1% of the participants reported insomnia/poor sleep, of which 50.3% slept <6 h. There are 818 CVD events. There was a higher risk of incident CVD in the insomnia/poor sleep with short sleep group HR: 1.29 (95% CI: 1.00-1.66), but sleep duration only was not associated with higher incidence of CVD.	Insomnia/poor sleep with short sleep duration was associated with higher risk of CVD incident.	Propensity score adjusted
9	Yang et al. [26]	<6, 6-8, and >8 hours	6-8 hours	Coronary heart disease	2740 participants (4.52%) developed coronary heart disease. Participants in the group of <6 h sleep were significantly associated with an increased risk of CHD with HR 1.13 (95% CI: 0.98-1.26). No significant association in >8 h.	Shorter sleep duration was associated with a higher risk of coronary heart disease.	Age, sex, educational level, marital status, alcohol drinking, cigarette smoking, vegetable intake, fruit intake, physical activity in leisure time, physical activity in work, family history of cardiovascular disease, body mass index, total cholesterol, fasting glucose, triglyceride levels, and systolic blood pressure
10	Wannamethee et al. [28]	Normal stable (7.4 to 7.5 hours), normal decreasing (7.0 to 5.5 hours), low increasing (4.9 to 6.9 hours), and low stable (4.2 to 4.9 hours)	7.4 to 7.5 hours	Cardiovascular events (atrial fibrillation, myocardial infarction, and stroke)	2406 participants had CVE. Compared with the normal stable pattern and adjusting for potential confounders, a low-increasing pattern was associated with increased risk of first CVEs (hazard ratio (HR): 1.22; 95% CI: 1.04-1.43), a normal-decreasing pattern was associated with increased risk of all-cause mortality (HR: 1.34; 95% CI: 1.15-1.57), and the low-stable pattern was associated with the highest risk of CVEs (HR: 1.47; 95% CI: 1.05-2.05) and death (HR: 1.50; 95% CI: 1.07-2.10).	The low-stable pattern sleep was associated with the highest risk to develop CVE, followed by low-increasing pattern sleep.	Age, sex, marital status, occupation, mean income, educational attainment, physical activity, smoking status, alcohol consumption status, salt intake, family history of stroke, MI, hypertension, hyperlipidemia, diabetes, snoring frequency, sleep duration in 2010, antihypertensive use, hypoglycemic use, use of agents lowering lipid levels, body mass index, fasting blood glucose level, high-sensitivity C-reactive protein, systolic blood pressure, diastolic blood pressure, and estimated glomerular filtration rate

CHD = coronary heart disease; CI = confidence interval; CVD = cardiovascular disease; CVE = cardiovascular event; h = hours; HDL-c = high-density lipoprotein cholesterol; hs-CRP = high-sensitivity C-reactive protein; LDL-c = low-density lipoprotein cholesterol.
